# Enhancement of lipid peroxidation and its amelioration by vitamin E in a subject with mutations in the *SBP2* gene[S]

**DOI:** 10.1194/jlr.M059105

**Published:** 2015-11

**Authors:** Yoshiro Saito, Mototada Shichiri, Takashi Hamajima, Noriko Ishida, Yuichiro Mita, Shohei Nakao, Yoshihisa Hagihara, Yasukazu Yoshida, Kazuhiko Takahashi, Etsuo Niki, Noriko Noguchi

**Affiliations:** * Systems Life Sciences Laboratory, Department of Medical Life Systems, Faculty of Life and Medical Sciences, Doshisha University, Kyotanabe, Kyoto 610-0394, Japan; † Health Research Institute, National Institute of Advanced Industrial Science and Technology (AIST), Ikeda, Osaka 563-8577, Japan; § Department of Pediatric Endocrinology and Metabolism, Aichi Children’s Health and Medical Center, Obu, Aichi 474-8710, Japan; ** Department of Nutritional Biochemistry, Hokkaido Pharmaceutical University, Otaru, Hokkaido 047-0264, Japan

**Keywords:** selenocysteine insertion sequence-binding protein 2, selenoprotein, antioxidative defense, free radical, cholesterol, oxysterol, oxidized lipids

## Abstract

Selenocysteine (Sec) insertion sequence-binding protein 2 (SBP2) is essential for the biosynthesis of Sec-containing proteins, termed selenoproteins. Subjects with mutations in the *SBP2* gene have decreased levels of several selenoproteins, resulting in a complex phenotype. Selenoproteins play a significant role in antioxidative defense, and deficiencies in these proteins can lead to increased oxidative stress. However, lipid peroxidation and the effects of antioxidants in subjects with *SBP2* gene mutations have not been studied. In the present study, we evaluated the lipid peroxidation products in the blood of a subject (the proband) with mutations in the *SBP2* gene. We found that the proband had higher levels of free radical-mediated lipid peroxidation products, such as 7β-hydroxycholesterol, than the control subjects. Treatment of the proband with vitamin E (α-tocopherol acetate, 100 mg/day), a lipid-soluble antioxidant, for 2 years reduced lipid peroxidation product levels to those of control subjects. Withdrawal of vitamin E treatment for 7 months resulted in an increase in lipid peroxidation products. Collectively, these results clearly indicate that free radical-mediated oxidative stress is increased in the subject with *SBP2* gene mutations and that vitamin E treatment effectively inhibits the generation of lipid peroxidation products.

Selenium is primarily incorporated into proteins in the form of selenocysteine (Sec). Sec is the 21st amino acid to be translated and is encoded by the UGA codon (1). The biological role of selenium is mediated through Sec-containing proteins, termed selenoproteins (2, 3). A stem-loop RNA structure, called the Sec insertion sequence (SECIS), is located in the 3′-untranslated region of selenoprotein mRNAs and is essential for the incorporation of Sec during the biosynthesis of selenoproteins (4). The SECIS interacts with a multi-protein complex, including SECIS-binding protein 2 (SBP2), which promotes Sec incorporation into growing polypeptides through selenocysteyl-transfer RNA at the UGA codon (4, 5). Although UGA primarily encodes a stop codon, this protein complex ensures that UGA is translated to Sec. The interaction between the SECIS element and SBP2 is particularly important for this translational system. Thus, defects in *SBP2* result in decreased levels of several selenoproteins (6–8). To date, nine families with *SBP2* mutations have been discovered (6–8). Twenty-five genes encoding selenoproteins have been identified in the human genome and have been shown to play diverse physiological roles, although the functions of many selenoproteins are not known (3, 9). The iodothyronine deiodinases are selenoproteins that regulate thyroid hormone action (10). Patients who harbor mutations in *SBP2* have characteristic abnormalities in thyroid hormone levels [high thyroxine (T_4_), low triiodothyronine (T_3_), and normal or slightly elevated thyroid-stimulating hormone (TSH)] and phenotypes including short stature during childhood and bone maturation delay due to the decrease in the iodothyronine deiodinases (6–8). Administration of the active form of thyroid hormone, T_3_, has been demonstrated to improve these symptoms (7, 8, 11). Furthermore, several selenoproteins, including glutathione peroxidases (GPxs), function to remove hydroperoxides, thereby preventing oxidative stress (12, 13). In humans, there are five GPx isoforms, namely GPx1–GPx4 and GPx6, which are selenoproteins with a Sec in the catalytic center. These isoforms differ in many properties, including their localization, subunit structure, primary structure, and enzymatic nature. Notably, the phospholipid hydroperoxide GPx (PHGPx, also called GPx4) plays a unique role in reducing a variety of hydroperoxides, including lipid hydroperoxides (e.g., phosphatidylcholine and cholesterol hydroperoxides) (14). It is well-established that PHGPx is the only GPx that can reduce cholesterol hydroperoxide. Deficiencies in selenoproteins can lead to increased lipid peroxidation and oxidative stress. Subjects with mutations in the *SBP2* gene show a complex phenotype related to oxidative stress, including photosensitivity and azoospermia (6–8). In addition, a previous study reported that red blood cell (RBC) and lymphocyte counts are slightly reduced in patients with *SBP2* mutations (7). The antioxidant defense systems of peripheral blood mononucleated cells and RBCs are also reported to be reduced (7). These lines of evidence strongly suggest that lipid peroxidation and oxidative stress are elevated in subjects with *SBP2* mutations, resulting in several disorders, such as low blood cell counts, photosensitivity, and azoospermia. However, the details of lipid peroxidation and the effects of antioxidant treatments have not been investigated.

The products of lipid peroxidation have been well-described (15). Polyunsaturated fatty acids and cholesterol, as well as their esters, are vulnerable to oxidation (15, 16). Polyunsaturated fatty acids and cholesterol are oxidized both enzymatically and nonenzymatically to produce several types of oxidation products. Free radical-mediated lipid peroxidation generates specific products; therefore, the progression as well as prevention of free radical-mediated lipid peroxidation could be evaluated by measuring these specific products (17, 18). In the case of linoleate, enzymatic oxidation by 12/15-lipoxygenase results in the formation of 13*S*-hydroperoxy-9*Z*,11*E*-octadecadienoic acid (13S-[*Z*,*E*]HPODE) exclusively; whereas, (*Z*,*E*)-HPODEs are generated by singlet oxygen, a non-free radical reactive oxygen species. Free radical oxidation of linoleate induces the formation of all HPODE isomers, including 9- and 13-(*Z*,*E*)- and (*E*,*E*)-HPODEs. HPODEs are readily reduced in vivo by reducing enzymes such as GPxs, thereby producing HODEs. Therefore, 9- and 13-(*E*,*E*)-HODEs are a marker of free radical-mediated oxidation of linoleate and can be assessed through reduction with triphenylphosphine and saponification with potassium hydroxide (19, 20). Free radical-mediated oxidation of arachidonate produces numerous isomers of F_2_-isoprostanes (21). In these isomers, 8-iso-prostaglandin F_2_α (isoP) has been demonstrated as a specific product of free radical-mediated oxidation of arachidonate (21, 22). In the case of cholesterol, free radical-mediated oxidation induces the formation of 7α- and 7β-hydroperoxycholesterol (7α- and 7β-OOHCh) (16, 18). The 7-OOHChs are reduced by PHGPx, thereby producing 7α- and 7β-hydroxycholesterol (7α- and 7β-OHCh). Enzymatic oxidation of cholesterol results in the formation of 7α-OHCh exclusively. Therefore, 7β-OHCh is determined as a marker of free radical-mediated oxidation of cholesterol after reduction and saponification (16, 18). Structures of the free radical-mediated lipid peroxidation products focused on this manuscript are shown in **Fig. 1**.

**Fig. 1.**
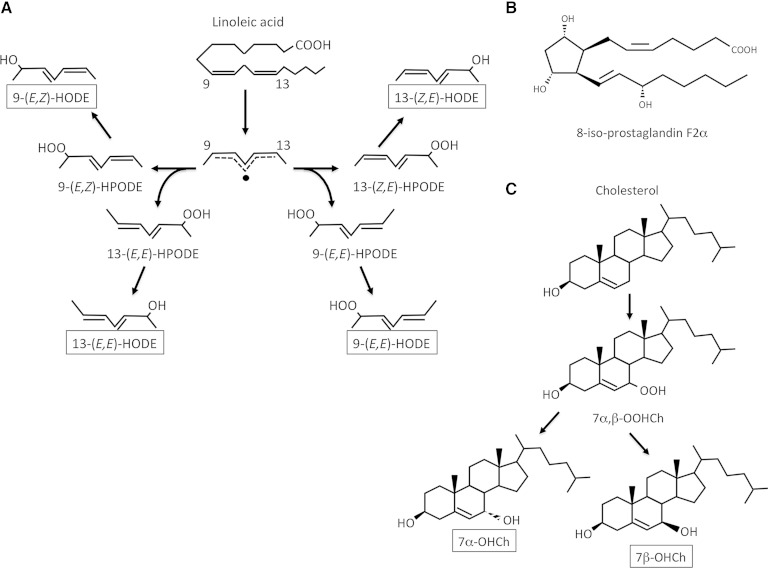
The structure of lipid peroxidation products studied in this work. A: Reaction pathways of the peroxidation of linoleate. B: Structure of 8-iso-prostaglandin F2α. C: Reaction pathways of the peroxidation of cholesterol.

α-Tocopherol (α-T), a primary form of vitamin E in vivo, is a potent lipid-soluble antioxidant (17, 23). The acetated form of α-T, namely α-T acetate, has been used as a pharmaceutical agent for the treatment of several diseases (23, 24). Previous studies have demonstrated that disorders induced by selenium deficiency or genetic knockdown of PHGPx are rescued by the administration of vitamin E (25–29). Recently, PHGPx has been identified as an essential regulator of ferroptosis, an iron-dependent form of nonapoptotic cell death discovered by Stockwell’s research group, which is also inhibited by free radical scavenging antioxidants, including vitamin E (30, 31). Therefore, vitamin E can be considered a reasonable antioxidant for subjects with *SBP2* mutations.

In the present study, we determined the levels of lipid peroxidation products in a subject with compound heterozygous mutations in the *SBP2* gene, which was previously identified and reported by our research group (8). This is the ninth patient to be reported in the world. We determined the levels of lipid peroxidation products, blood cells, and biochemical profiles, and examined the effects of vitamin E treatment on the levels of these biomarkers.

## MATERIALS AND METHODS

### Subjects

All experiments were performed in accordance with relevant guidelines and regulations. All procedures were approved by the Ethics Committee of Aichi Children’s Health and Medical Center, Doshisha University, and the National Institute of Advanced Industrial Science and Technology. Written informed consent for molecular studies, T_3_ treatment, and vitamin E treatment was obtained from the patient’s parents. For T_3_ treatment, liothyronine sodium (thyronamine; Takeda Pharmaceutical Co. Ltd., Osaka, Japan; 5 μg/day) was given in two divided doses. In the case of vitamin E treatment, α-T acetate [Juvela tablets (50 mg); Eisai Co. Ltd., Tokyo, Japan; 100 mg/day] was given in two divided doses. The proband (a 10-year-old boy), his parents, and two brothers were recruited to this study. The proband was treated at the Aichi Children’s Health and Medical Center. Six control subjects treated at the same institute were also recruited. The basic information and observed disorders of the proband and control subjects are summarized in **Table 1**. Serum was obtained by centrifugation at 3,000 *g* for 5 min at 4°C and was stored at −80°C. For plasma preparation, blood was collected in ethylenediaminetetraacetic acid-containing tubes, and centrifuged at 3,000 *g* for 10 min at 4°C. The plasma was also stored at −80°C prior to analysis.

**TABLE 1.** The information of the proband and control subjects recruited to this study

	Age (years)	Sex	Basic Disorder
Proband	10	M	*SBP2* gene mutations
Average of control*^a^*	11 (2)	—	—
Control 1	9	M	Precocious puberty
Control 2	10	F	Idiopathic short stature
Control 3	13	M	Idiopathic short stature
Control 4	10	M	Precocious puberty
Control 5	13	M	Idiopathic short stature
Control 6	12	M	Idiopathic short stature

a Mean value is shown with standard deviation in parentheses.

### Western blot analysis

**Fig. 2.**
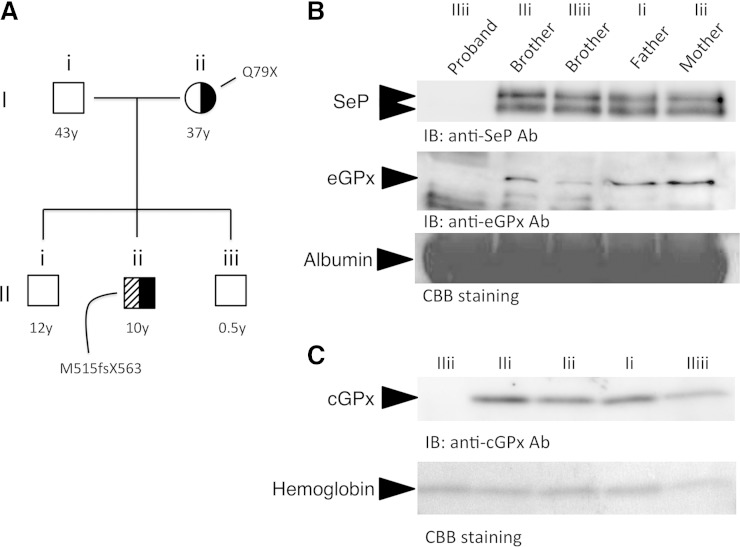
Blood selenoproteins are reduced in the proband. A: Pedigree of the proband’s family with mutations in *SBP2*. Squares and circles represent male and female family members, respectively, and filled symbols denote the mutation in *SBP2*. B: Western blot analysis of serum selenoproteins. Upper panel: Immunoprecipitation and Western blot analysis of SeP. Proteins were immunoprecipitated from each serum sample (1 μl) using an anti-human SeP mAb (clone BD1, 2 μg) conjugated to magnetic beads, followed by Western blot analysis. Proteins in the membrane were detected using an anti-human SeP mAb (clone BD3, 1 μg/ml). Middle panel: Western blot analysis of eGPx. Each serum sample (1 μl) was subjected to Western blot analysis using an anti-human eGPx polyclonal Ab (pAb) (5 μg/ml). Lower panel: As a loading control, separated proteins were stained with CBB. The major band derived from albumin is indicated. C: Upper panel: Western blot analysis of cGPx in RBCs. Cell lysates (200 μg for Western blot analysis) obtained from RBCs were subjected to Western blot analyses using an anti-cGPx pAb (1 μg/ml). Lower panel: As a loading control, separated proteins were stained with CBB. The major band derived from hemoglobin is indicated.

Preparation of the whole-cell extracts and Western blot analysis were conducted as described previously (32, 33). Immunoprecipitation assays were conducted using the rat anti-human selenoprotein P (SeP) monoclonal Abs (mAbs), BD1 and BD3 (34). BD1 mAb (2 μg) was coupled to Dynabeads Protein G (20 μl, Invitrogen Life Technologies, Carlsbad, CA) using a chemical cross-linker, dimethyl pimelimidate (Thermo Fisher Scientific Inc., Waltham, MA). The BD1 mAb-conjugated Dynabeads (20 μl) were then applied to human serum (1 μl) and incubated for 1 h at 4°C. The beads were washed, and eluted protein samples were subjected to Western blot analysis, as described below. For Western blot analysis, rat anti-human SeP mAb [BD3 (34), 1 μg/ml], chicken anti-human extracellular GPx (eGPx) Ab [(35), 5 μg/ml], and rabbit anti-cellular GPx (cGPx) Ab (1 μg/ml, LF-PA0019; Lab Frontier Co. Ltd., Seoul, Korea) were used. As a loading control for the serum samples, separated proteins were stained with Coomassie Brilliant Blue R-250 (CBB). The major band derived from each serum sample is indicated in **Fig. 2**.

### Determination of lipid peroxidation products

The oxidation products of linoleates, arachidonates, and cholesterol were measured as free fatty acid hydroxides, free isoP, and free hydroxycholesterol, respectively, as described in the previous report with slight modification (20). Briefly, the serum or plasma was mixed with the internal standards, 13-HODE-d4, 7β-OHCh-d7, and 8-isoP-d4, and butylated hydroxytoluene was added to the samples. This was followed by the reduction of hydroperoxides using 1 mM triphenylphosphine (Sigma-Aldrich, St. Louis, MO) at room temperature for 30 min, followed by saponification with 1 M KOH. Lipids were extracted, and then divided equally into two portions: a sample for LC-MS/MS and a sample for GC-MS. The levels of 7β-OHCh, total cholesterol, and total linoleic acid were measured by GC-MS, while the levels of HODE were measured by LC-MS/MS using a previously reported method (20).

### Determination of vitamin E content

Serum vitamin E concentration was measured using a high-performance LC system with electrochemical detection as described previously (36).

#### Other clinical tests.

Blood cells were analyzed by flow cytometry (XE-5000; Sysmex Corporation, Kobe, Japan). Serum aspartate aminotransferase (AST) and alanine aminotransferase (ALT) were determined by the Japan Society of Clinical Chemistry standardized method (L Type Wako AST/ALT J2 kit; Wako Pure Chemical Industries, Ltd., Osaka, Japan). Blood glucose was determined by using the hexokinase glucose-6-phosphate dehydrogenase method (L Type Wako Glu2 kit; Wako Pure Chemical Industries, Ltd.). Chemiluminescence enzyme immunoassays (Lumipulse TSH-III, free T_3_-N, and free T_4_; Fujirebio Inc., Tokyo, Japan) were used to measure TSH, free T_3_, and free T_4_.

### Statistical analysis

The statistical significance of the difference between determinations was calculated using the Student’s *t*-test and ANOVA using Tukey test for multiple comparisons. Values of *P* < 0.01 were considered significant.

## RESULTS

### Selenoprotein contents in the blood of the subject with *SBP2* mutations and unaffected family members

We previously identified and reported a subject (the proband) harboring *SBP2* mutations (8). The pedigree of the proband’s family is shown in Fig. 2A. In the proband, compound heterozygous mutations (p.M515fsX563/p.Q79X) have been identified. In the present study, Western blot analysis of serum selenoproteins, such as SeP and eGPx, was performed. Serum levels of SeP and eGPx in the proband were extremely low (Fig. 2B). A Western blot analysis of cGPx in RBCs was also conducted, which demonstrated that cGPx in RBCs was also markedly decreased in the patient (Fig. 2C). The levels of blood selenoproteins in the proband’s younger brother (IIiii), a 6-month-old baby, were slightly lower. The *SBP2* gene of the brother showed no mutations (Fig. 2A), and levels of these selenoproteins were detectable. Therefore, we speculate that lower levels of these selenoproteins in the younger brother (IIiii) are the result of age-related effects. Collectively, these data indicate that the subject with *SBP2* mutations is markedly selenoprotein deficient.

### Evaluation of lipid peroxidation products in the serum of the proband and control subjects

To evaluate lipid peroxidation, the oxidation products of lipids in the serum and plasma of the subject with *SBP2* mutations and control subjects were analyzed. Six control individuals treated at the same institute as the proband were recruited to the present study because they did not show noticeable symptoms, such as a hormone disorder or skeletal dysplasia. The basic information and observed disorders of the control subjects are summarized in Table 1. Lipids in serum and plasma were reduced by triphenylphosphine, followed by saponification, and then measured by LC-MS/MS and GC-MS, as described previously (20). By using this method, the oxidation products of linoleates, arachidonates, and cholesterol were measured as free HODEs, free isoP, and free OHCh, respectively. In the present study, we assessed the lipid peroxidation products in both the plasma and serum, and confirmed that both determinants showed similar levels at some time-points.

**Fig. 3.**
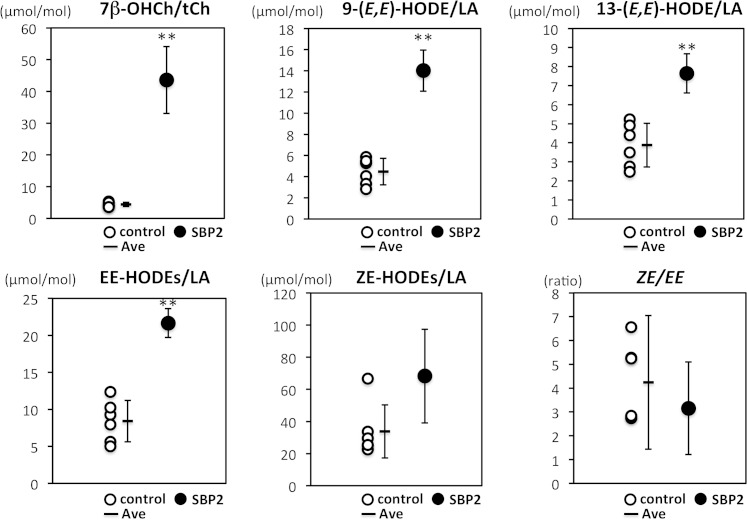
Lipid peroxidation products are elevated in the serum of the *SBP2* mutant. Serum lipid peroxidation products were measured as described in the Materials and Methods. The mean values ± SD in the proband (n = 4) and control subjects (n = 6) are shown with as individual dots. **P < 0.01 (Student’s *t*-test) when compared with control subjects.

The results are shown in **Fig. 3** and **Table 2**. We found markedly high levels of the free radical-mediated oxidation product of cholesterol, 7β-OHCh, in this patient (Fig. 3). This result suggests that there is an increase in free radical-mediated lipid peroxidation in the subject harboring *SBP2* mutations. In the case of linoleates, the levels of free radical-mediated oxidation products, 9-(*E*,*E*)-HODE and 13-*(E*,*E*)-HODE, in the proband were higher than those of the control subjects. EE-HODEs [the sum of 9-(*E*,*E*)- and 13-(*E*,*E*)-HODE] in the proband were also calculated to be elevated (Fig. 3). These free radical-mediated oxidation products showed a significant increase in the subject with *SBP2* mutations when compared with the mean values of multiple control samples. By contrast, in the case of ZE-HODEs [the sum of 9-(*E*,*Z*)- and 13-(*Z*,*E*)-HODE], which are not free radical-specific peroxidation products, these levels were not significantly higher in the proband than those of the control group (Fig. 3, Table 2). This method of measurement can obtain qualitative and quantitative information on the generation of oxidized linoleic acid, and measurement of the stereoisomer ratio (ZE-HODEs/EE-HODEs) can be used to determine the efficacy of the antioxidants in vivo (37); however, this ratio was not significantly changed (Fig. 3). The levels of linoleate and cholesterol were also not altered (Table 2). Collectively, our data suggest that there is an increase in free radical-mediated lipid peroxidation, in particular in cholesterol oxidation, in the subject harboring *SBP2* mutations.

**TABLE 2.** Lipid peroxidation products in the serum of control subjects and the proband with or without α-T treatment

	Proband			
	Before α-T	α-T for 24M	Withdrawal for 7M	Control (n=6)
HODE/LA (μmol/mol)				
9EE	14 (1.9)*^a^*	2.5 (0.1)*^b^*	9.1 (1.4)	4.5 (1.3)
9ZE	37 (20)	14 (3)	15 (6)	17 (11)
13EE	7.6 (1.0)*^a^*	1.8 (0.2)*^b^*	6.5 (0.7)	3.9 (1.1)
13ZE	33 (22)	12 (4)	19 (3)	17 (5.8)
tHODE	92 (43)	28 (6)	46 (4)	42 (18)
EE-HODE	22 (2.0)*^a^*	4.4 (0.1)*^b^*	15 (2.0)*^b^*	8.4 (2.8)
ZE-HODE	70 (29)	24 (6)*^b^*	31 (3)	34 (17)
ZE/EE (ratio)	3.2 (1.3)	5.4 (1.2)	2.1 (0.1)	4.0 (1.7)
Linoleate (mM)	1.3 (0.1)	1.6 (0.1)	1.8 (0.1)	2.0 (0.7)
7β-OHCh/Ch (μmol/mol)	44 (11)*^a^*	5.4 (0.7)*^b^*	7.0 (0.5)*^b^*	4.4 (0.7)
Ch (mM)	3.7 (0.1)	3.6 (0.5)	4.0 (0.5)	4.3 (0.6)
IsoP (pM)	50 (15)	30 (35)	19 (7)	100 (130)
α-T (μM)	8.4 (0.4)	15 (0.4)*^b^*	9.5 (0.3)	11 (1.9)
γ-T (μM)	4.1 (0.5)	0.73 (0.03)*^b^*	3.9 (0.01)	4.4 (2.1)

The serum lipid peroxidation products were measured as described in the Materials and Methods. The mean values in the proband (n = 3–4) and control subjects (n = 6) are shown with standard deviation in parentheses.

a *P* < 0.01 (Student’s *t*-test) when compared with control subjects.

b *P* < 0.01 (Tukey, ANOVA) when compared with before α-T treatment.

### Serum content of α- and γ-T in the subject with *SBP2* mutations, and the effects of α-T acetate administration

**Fig. 4.**
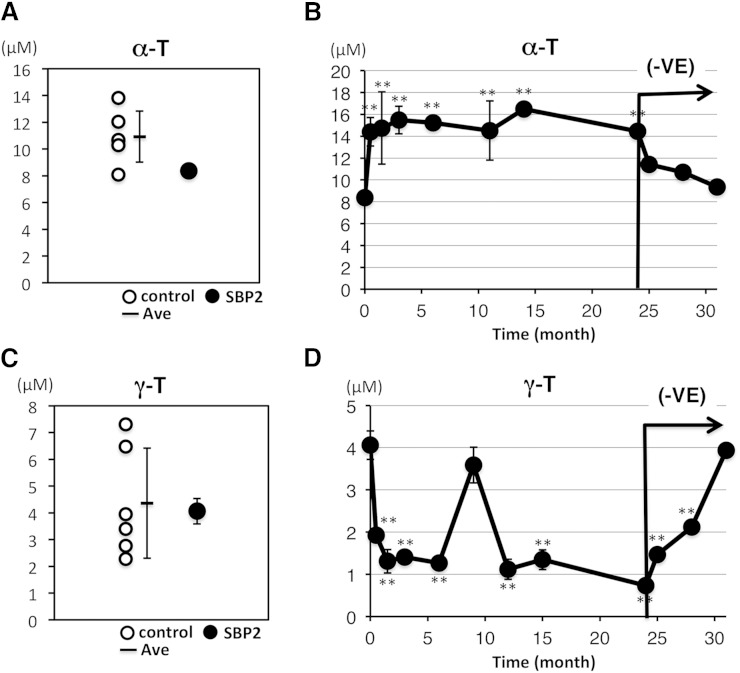
Analysis of α- and γ-T levels in the serum of the *SBP2* mutant and control subjects and the effect of vitamin E treatment and withdrawal. A, C: Serum α- and γ-T levels were measured as described in the Materials and Methods. The mean values ± SD in the proband (n = 4) and control subjects (n = 6) are shown as individual dots. B, D: The alteration of α-T and γ-T levels depending on vitamin E treatment and its withdrawal. The subject received 100 mg/day α-T acetate for 2 years, followed by withdrawal of treatment for 7 months. During this time, α-T and γ-T levels were assessed, and the mean values ± SD (n = 3–4) were plotted against time. The duration of vitamin E withdrawal is indicated as (−VE). **P < 0.01 (Tukey, ANOVA) when compared with time 0.

The serum content of α-T, a major lipid-soluble antioxidant, in the proband was determined and compared with those of control subjects. The serum level of α-T in the subject with *SBP2* mutations tended to be low compared with those of the control subjects (**Fig. 4A**, Table 2). Next, we examined the effects of vitamin E treatment on the proband by monitoring α-T levels and biomarkers of oxidative stress. α-T acetate (100 mg/day) was administered to the proband for 2 years. The time-dependent change of serum α-T content is shown in Fig. 4B. The administration of α-T acetate increased serum levels of α-T, and the elevated levels of α-T were sustained for 2 years. Serum levels of α-T started to decrease after withdrawal of vitamin E treatment and returned to their original levels in 7 months. In the present study, we also determined γ-T, the second-highest isoform of vitamin E in human serum. The level of γ-T in the proband was similar to those of control subjects (Fig. 4C, Table 2). The administration of α-T acetate decreased serum levels of γ-T, and the decreased levels were sustained for 2 years (Fig. 4D). Serum levels of γ-T started to increase after withdrawal of vitamin E treatment and returned to the original levels in 7 months (Fig. 4D).

Administration of the active form of thyroid hormone, T_3_, has been demonstrated to improve symptoms such as short stature during childhood and bone maturation delay (7, 8). The administration of T_3_ (5 μg/day) to the proband was simultaneously started with vitamin E treatment and continued until the end of the study. The administration of T_3_ increased free T_3_ levels and decreased free T_4_ levels during the study (**Table 3**, supplementary Fig. 1).

**TABLE 3.** Blood cells and biochemical profiles of control subjects and the proband with or without α-T treatment

	Proband			Control (n=6) Normal range*^b^*
	Before α-T	α-T for 24M	Withdrawal for 7M	
WBC (×10/mm^3^)	486 (53)	872	445	563 (37) 450–1350
Neutro (×10/mm^3^)	243 (17)	704	220	267 (76) 300–580
Lymph (×10/mm^3^)	184 (25)	102	177	237 (59) 150–300
RBC (×10^4^/mm^3^)	462 (29)	489	487	458 (31) 400–520
PLT (×10^4^/mm^3^)	27 (2)	26	24	26 (4) 15–40
HB (g/dl)	13 (1)	14	14	13 (1) 12–16
HCT (%)	38 (2)	40	40	38 (2) 35–45
AST (IU/l)	27 (2)	22	22	24 (3) 8–38
ALT (IU/l)	17 (5)	16	18	13 (2) 4–44
BS (mg/dl)	78 (16)	96	94	95 (9) 70–110
TSH (μU/ml)	2.2 (0.3)	1.9	0.7	1.4 (0.4) 0.5–3.7
FT_3_ (pg/ml)	2.3 (0.3)	4.2	3.4	3.9 (0.4) 2.5–4.1
FT_4_ (pg/ml)	2.3 (0.1)*^a^*	1.7	1.9	1.0 (0.2) 0.9–1.5

These values were determined as described in the Materials and Methods. The mean values in the proband (before α-T treatment, n = 3) and control subjects (n = 6) are shown with standard deviation in parentheses. In the other points, representative values are shown. WBC, white blood cells; Neutro, neutrophils; Lymph, lymphocyte; HB, hemoglobin; HCT, hematocrit; PLT, platelet; BS, blood sugar; FT_3_, free T_3_; FT_4_, free T_4_.

a *P* < 0.01 (Student’s *t*-test) when compared with control subjects.

b Normal values for WBC, Neutro, Lymph, RBC, PLT, HB, and HCT were referenced from (55). Other values were referenced from each kit used for measurement.

### The effects of vitamin E treatment on lipid peroxidation products

**Fig. 5.**
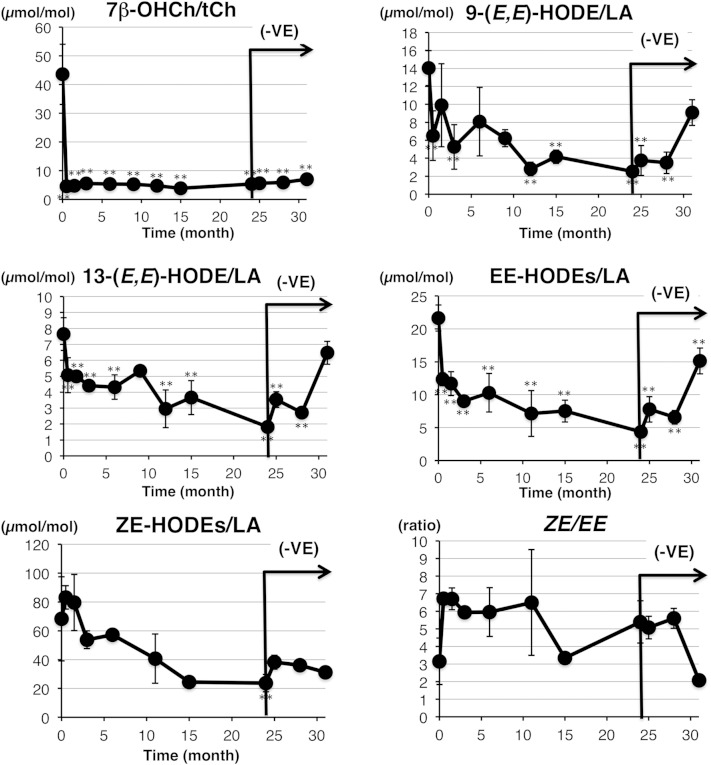
Elevated levels of lipid peroxidation products in the serum of the *SBP2* mutant are corrected by vitamin E supplementation. Lipid peroxidation products were determined over the course of 2 years of vitamin E treatment and 7 months of withdrawal, and the mean values ± SD (n = 3–4) were plotted against time. The oxidized lipids analyzed are shown at the top of each graph. The duration of vitamin E withdrawal is indicated as (−VE). **P < 0.01 (Tukey, ANOVA) when compared with time 0.

Next, we measured lipid peroxidation products to evaluate the effects of vitamin E treatment. Vitamin E treatment effectively decreased the levels of lipid peroxidation products in the serum of the subject harboring *SBP2* mutations (**Fig. 5**). Vitamin E treatment for only a 2 week period was sufficient to dramatically decrease 7β-OHCh levels, an effect that also persisted for 2 years (Fig. 5). The levels of 7β-OHCh did not increase after the withdrawal of vitamin E treatment. On the other hand, free radical-specific oxidation products of linoleates, such as 9-(*E*,*E*)-, 13-(*E*,*E*)-, and EE-HODEs, were decreased by vitamin E treatment for 2 years. Free radical-specific HODEs increased dramatically after the withdrawal of vitamin E treatment (Fig. 5). In contrast, a similar change was not observed in ZE-HODEs and the stereo-isomer ratio (*ZE*/*EE*) (Fig. 5). Collectively, these results indicate that vitamin E treatment effectively suppressed free radical-mediated lipid peroxidation in the subject with *SBP2* mutations.

### The effects of vitamin E treatment on white blood cell count

**Fig. 6.**
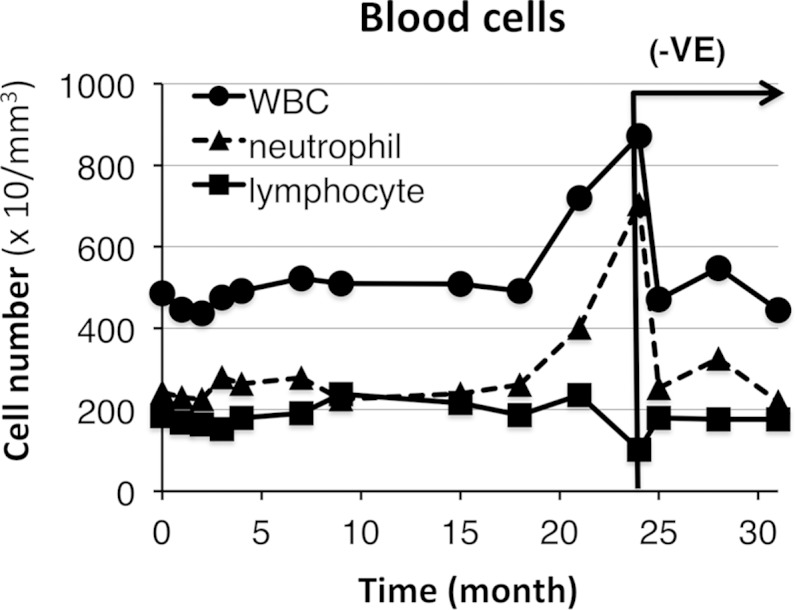
Isolated neutropenia and lymphocytopenia in the *SBP2* mutant are rescued by vitamin E treatment. The number of white blood cells (WBC), neutrophils, and lymphocytes were determined over the course of 2 years of vitamin E treatment and 7 months of withdrawal and plotted against time. The duration of vitamin E withdrawal is indicated as (−VE).

RBC and lymphocyte counts have been previously reported to be slightly reduced in the subjects with *SBP2* mutations (7). In the present study, we also determined the blood cell count in the proband and control subjects. These values are summarized in Table 3. The levels of blood cells, such as white blood cells and lymphocytes, in the proband tended to be reduced compared with those of the control subjects. White blood cell levels gradually increased during vitamin E treatment of the proband and immediately decreased after withdrawal of treatment (**Fig. 6**). The number of neutrophils also exhibited a similar tendency to that of white blood cell levels (Fig. 6). These results suggest that vitamin E treatment affected the number of neutrophils in the proband.

To determine the functioning of the liver and glucose metabolism in the proband, serum AST, ALT, and blood glucose were determined. As shown in Table 3, these values were within the normal range, and vitamin E treatment did not cause obvious changes to these levels.

## DISCUSSION

SBP2 plays a significant role in the synthesis of selenoproteins, and defects in its gene decrease selenoprotein levels, resulting in multiple disorders (6–8). Some selenoproteins play an important role in antioxidative defense. Indeed, Schoenmakers et al. (7) have reported the elevation of reactive oxygen species in RBCs of subjects with *SBP2* mutations by using a fluorescent probe, 3′-(p-aminophenyl)fluorescein. In this previous report, in vitro experiments also suggested that the antioxidant vitamin E could suppress UV-induced oxidative stress in the fibroblasts of affected subjects. The administration of antioxidants to these patients is expected to be useful; however, the details of the beneficial effects of antioxidant treatments have not been established. To better understand whether antioxidant therapies are useful for patients with *SBP2* mutations, two important issues must be addressed: what are reliable biomarkers for monitoring oxidative damage and which antioxidants are suitable for treatment of oxidative damage?

In the present study, we focused on the lipid peroxidation products of linoleates and cholesterol because their oxidation gives rise to simpler products than highly unsaturated lipids, such as arachidonates (15, 16). As shown in Fig. 5, vitamin E treatment obviously decreased the levels of free radical-mediated lipid peroxidation products, and its withdrawal increased these levels. Vitamin E is the most abundant lipophilic radical-scavenging antioxidant. Based on the kinetic data and physiological molar ratio of vitamin E to substrates, the peroxyl radicals are the only radicals that vitamin E can scavenge efficiently in vivo (38). Collectively, our results and previous reports strongly indicate that the enhancement of free radical-mediated lipid peroxidation in the subject with *SBP2* mutations is suppressed by vitamin E treatment. Cholesterol oxidation products are well-known to be toxic to cells and to cause unfavorable effects on the body. Cholesterol oxidation products are believed to relate to several symptoms involving oxidative stress, such as photosensitivity and azoospermia, in patients with *SBP2* mutations via free radical-mediated lipid peroxidation (6–8). In addition, we have previously reported the role of cholesterol oxidation on Jurkat cell death induced by selenium deficiency (25). Therefore, the generation of oxidized cholesterol is believed to affect the proliferation of white blood cells. Vitamin E treatment is considered to be effective to treat these symptoms in *SBP2* mutants via the suppression of free radical-mediated cholesterol oxidation.

Notably, our study indicates that the level of 7β-OHCh in the subject was remarkably high and that vitamin E treatment dramatically decreased 7β-OHCh levels (Figs. 3, 5). Cholesterol and linoleate are frequently the targets of free radicals, and the chemical reactivity of these lipids changes depending on the milieu where the substrates exist (39). Indeed, linoleates and cholesterol oxidation were markedly different when comparing plasma and cellular lipid peroxidation (18, 40). During plasma lipid peroxidation, linoleates were preferentially oxidized, inducing the formation of HODEs (18, 39). In contrast, cellular lipid peroxidation preferentially oxidized cholesterol over linoleates (18, 40). Additionally, previous studies have shown the preferential oxidation of cholesterol to form 7β-OHCh in selenium-deficient Jurkat cells (25, 40). Thus, it is interesting to hypothesize that higher levels of 7β-OHCh in the subject reflect cellular lipid peroxidation. Notably, the effects of vitamin E withdrawal on the levels of 7β-OHCh were small compared with those of the oxidized products of linoleates. The tissue contents of vitamin E are determined by a balance between incorporation and elimination, and the rate of elimination of vitamin E from adipose tissue is slower than that from blood (23, 24). Taken together with the previous reports and our hypothesis, the lack of obvious effects of vitamin E cessation on 7β-OHCh levels appears to result from the difference in the elimination rate of vitamin E from tissues versus blood. The site of linoleate and cholesterol oxidation cannot be determined using the data in the present study. A study using tissue-specific *SBP2* knockout mice might help to elucidate the site and mechanisms of cholesterol and linoleate oxidation in the subject with *SBP2* mutations.

As shown in Fig. 4D, the administration of vitamin E altered γ-T content. Vitamin E content in serum is regulated by α-T transfer protein, which facilitates α-T transfer to the ATP binding cassette transporter A1 in the hepatic plasma membrane, resulting in the acceleration of α-T secretion to the plasma (41, 42). α-T transfer protein possesses a higher affinity for α-T than γ-T (41, 43). Therefore, we believe that the administration of α-T acetate resulted in the preferential binding of α-T to α-T transfer protein and decreased binding of γ-T and its serum content. Additionally, high α-T administration (400 IU) upregulates the xenobiotic metabolism mediated by cytochrome P 450 (CYP) such as CYP4F2 (44, 45). CYP4F2 activity toward α-T was limited relative to other forms of vitamin E (46). The half-life of γ-T is shorter than that of α-T (47). The upregulation of xenobiotic metabolism in the proband might also be related to the decrease in γ-T content induced by the administration of α-T acetate. Based on the measurements shown in Table 2, total vitamin E content in the proband increased from 13 to 17 μM by the administration of vitamin E. The relative reactivity of α-T toward free radicals is higher than that of γ-T (48). In addition, α- and γ-tocopheryl quinone, oxidized metabolites of α-T and γ-T, have different chemical and biological activity (49, 50). Thus, γ-T quinone has a property of being an arylating quinone, which forms covalently linked quinone-thiol Michael adducts and induces endoplasmic reticulum stress and adaptive response, while the nonarylating α-T quinone does not. These isoforms of vitamin E possess different properties; however, in the proband, the increase in α-T should be beneficial to suppress free radical-mediated lipid peroxidation.

In the clinical tests, the levels of white blood cells and neutrophils changed with the administration of vitamin E (Fig. 6, Table 3). RBC and lymphocyte counts have been reported to be slightly reduced in patients with *SBP2* mutations (7). In addition, the antioxidant defense systems of peripheral blood mononucleated cells and RBCs were reported as reduced (7). In the present study, the blood cell counts in this subject, particularly neutrophils and lymphocytes, were low, while RBC counts were slightly low, but within the normal range (Table 3). Vitamin E treatment increased the neutrophil numbers in the affected subject, and the vitamin’s withdrawal decreased neutrophil levels (Fig. 6). On the other hand, RBC counts were not affected (Table 3). Why vitamin E supplementation has effects on lymphocyte levels, but not on RBC counts, remains unclear at present. Neutrophil levels are regulated by several factors, including proliferation, differentiation, and movement from the bone marrow reserve (51). Nutrient deficiency, such as low vitamin B12 and folic acid, is known to decrease the blood levels of neutrophils (52). Selenium and vitamin E supplementation are reported to have beneficial effects on neutrophil-mediated bacterial death, but not on neutrophil phagocytosis (53). Collectively, these results suggest that vitamin E treatment is beneficial to enhance the immune function of patients with *SBP2* mutations via the increasing neutrophils. It is notable that a longer treatment time is needed to improve white blood cell counts (Fig. 6) compared with lipid oxidation products. The precise molecular mechanisms for the different effects of vitamin E on white blood cell counts and lipid oxidation are still unknown, but Salonen et al. (54) have reported that 6 year supplementation for men resulted in more beneficial effect for arteriosclerotic progression, as determined by common carotid artery intima-media thickness, than that of 3 year supplementation. A longer interval of supplementation with vitamin E appears beneficial for the immune function of patients with *SBP2* mutations via an increase in neutrophils.

Selenoenzyme PHGPx is the major GPx that reduces lipid-soluble hydroperoxides, including phospholipid hydroperoxides and cholesterol hydroperoxide (14). Several studies have demonstrated the important physiological role of PHGPx, and the rescue by vitamin E of several disorders induced by deficiency of PHGPx (25–31). Therefore, elevated levels of lipid peroxidation in this patient are thought to be primarily due to a deficiency in PHGPx. Vitamin E treatment from an early stage is expected to be profitable for several disorders related to lipid peroxidation in the subjects with *SBP2* mutations.

In conclusion, the present study clearly shows that free radical-mediated oxidative stress is enhanced in a subject with *SBP2* mutations and that vitamin E treatment is effective to inhibit the elevated lipid peroxidation. The effects of antioxidant supplementation, including by vitamin E, in many large-scale intervention studies, have shown disappointing results. To exert the beneficial effects of vitamin E on the inhibition of lipid peroxidation in various diseases involving free radical-mediated oxidative stress, treating the right subject at the right time and for the right duration might be important. Many of the functions of the identified 25 selenoproteins have not been elucidated yet, and their dysfunction is expected to contribute to the complex phenotype of *SBP2* mutations. Understanding the precise function of selenoproteins will help to achieve relevant care for subjects with *SBP2* mutations.

## Supplementary Material

Supplemental Data
