# Supplementary material for: Enhancement of lipid peroxidation and its amelioration by vitamin E in a subject with mutations in the SBP2 gene
Source: J Lipid Res. 2015 Nov;56(11):2172–82. doi: 10.1194/jlr.M059105 (PMC4617404; doi:10.1194/jlr.M059105)
Supplement: Supplemental Data [file supp_56_11_2172__index.html]

Enhancement of lipid peroxidation and its amelioration by vitamin E in a subject with mutations in the SBP2 gene — Enhancement of lipid peroxidation and its amelioration by vitamin E in a subject with mutations in the SBP2 gene — Supplemental Data 

# Enhancement of lipid peroxidation and its amelioration by vitamin E in a subject with mutations in the *SBP2* gene

## Supplemental Data

- Supplemental Fig (.pdf, 68 KB) - Supplemental Fig
