# Supplementary material for: Enhancement of lipid peroxidation and its amelioration by vitamin E in a subject with mutations in the SBP2 gene
Source: J Lipid Res. 2015 Nov;56(11):2172–82. doi: 10.1194/jlr.M059105 (PMC4617404; doi:10.1194/jlr.M059105)
Supplement: Supplemental Data [file 10.1194_M059105_jlr.M059105-1.pdf]

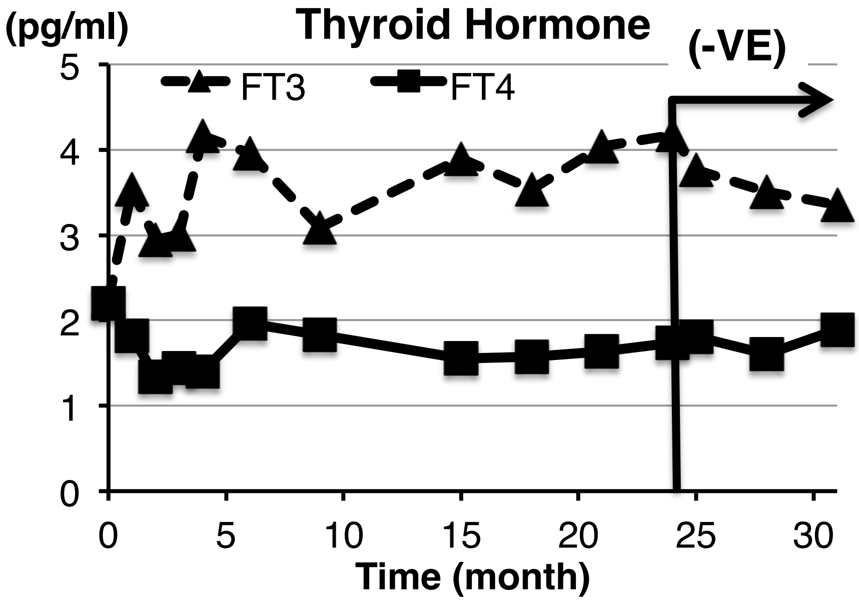

Supplemental Figure S1. The levels of thyroid hormone in the serum of the *SBP2* mutant over the course of the study. The administration of T_3 was simultaneously started with vitamin E treatment, and continued untill the end of the study (2 years and 7 months). During this time, the levels of free T_3 (FT_3) and free T_4 (FT_4) were determined and plotted against time.

Saito et al., Supplemental Figure S1
